# Using Distributed Temperature Sensing for Long-Term Monitoring of Pockmark Activity in the Gulf of Patras (Greece): Data Processing Hints and Preliminary Findings

**DOI:** 10.3390/s23208520

**Published:** 2023-10-17

**Authors:** Elias Fakiris, George Papatheodorou, Dimitris Christodoulou, Zafeiria Roumelioti, Efthimios Sokos, Maria Geraga, Vasileios Giannakopoulos, Xenophon Dimas, George Ferentinos

**Affiliations:** 1 Laboratory of Marine Geology and Physical Oceanography (Oceanus-Lab), Department of Geology, University of Patras, 26504 Patras, Greece; fakiris@upatras.gr (E.F.); dchristo@upatras.gr (D.C.); mgeraga@upatras.gr (M.G.); giannakopoulosv@upatras.gr (V.G.); xendimas@upatras.gr (X.D.); gferen@upatras.gr (G.F.); 2 Seismological Laboratory, Department of Geology, University of Patras, 26504 Patras, Greece; zroumelioti@upatras.gr (Z.R.); esokos@upatras.gr (E.S.)

**Keywords:** pockmark field activation, earthquakes, gas seepages, incomplete time series treatment, spectral analysis, Patras Gulf

## Abstract

Patras Gulf pockmark field (Western Greece) is a tectonically controlled field that has been activated at least twice by strong earthquakes (M5.4, 14 July 1993 and M6.4, 8 June 2008), and episodic gas seepages have been recorded in the past using geophysical means. A distributed temperature sensor (DTS) system was deployed inside a shallow pockmark and along an active fault at the northern end of the field. This ongoing experiment represents the first long-term monitoring ever conducted on gas-bearing pockmarks and active faults by the DTS system. For now, we have acquired and analyzed data regarding about 1.56 years. One of the primary objectives of this study is to establish methodological queues for data processing and analysis, including spectral analysis and incomplete data treatment techniques, to be standardized for use in further stages of the experiment. Spectral analysis was proven capable of separating the temperature footprint of background environmental components, such as sea-atmosphere heat flux, tides, and winds/waves, from high-frequency temperature residuals. Those residuals represent unusual events that might be correlated to seismicity. Monitoring the causal relationship between seismic activity and seabed water temperature changes in the field was thus attempted. No significant local earthquakes occurred during the monitoring period. Although the relation between seismicity and irregular seabed water temperature events was not systematic, we postulate that four thermal events have a causative link with the local seismicity. The DTS system constitutes a low-cost monitoring system, and the promising preliminary results of this experiment suggest that it is worth testing for a longer period.

## 1. Introduction

In recent years, the utilization of optical fiber technology has witnessed a remarkable upsurge across various applications due to its diverse capabilities, which extend to various domains, including the marine environment. A wide range of optical fiber sensing devices are utilized, with commercially available methods divided into two categories: point sensing, representing the earlier approach, and distributed sensing. Point sensing utilizes a restricted active fiber section of less than 2 cm, while distributed sensing uses the full length of the fiber as the sensing element, reaching up to tens of kilometers [1]. These sensors are resistant to electromagnetic interference and exceptionally durable in extreme conditions, such as those encountered in marine environments. With lightweight construction and high transmission rates, optical fiber sensors enable easy deployment, efficient data transfer, and monitoring of various parameters in the ocean. Additionally, an added advantage of optical fiber sensors is their ability to provide real-time, in situ measurements [2]. Distributed sensing, unlike single-point fiber-optic techniques such as fiber Bragg gratings, employs temperature-dependent light scattering in the Rayleigh, Brillouin, or Raman regimes within the fiber-optic core. This, combined with optical time domain reflectometry, enables uninterrupted temperature measurements along the longitudinal axis [3], thus providing large-scale monitoring [4]. Distributed temperature sensors based on Raman scattering (DTS-R) methods are frequently grounded in the time-domain optical reflectometry (OTDR) principle. This approach involves the emission of short pulses into the optical fiber, and by analyzing the discrepancy between the sending and returning light times, it is possible to ascertain the spatial distribution of temperature variations along the entire fiber [5]. By utilizing fiber-optic cables originally installed for communication purposes, it becomes cost-effective to acquire spatially dense and temporally continuous measurements by merely connecting an interrogator, which is a laser transmitter/receiver system, to one end of the cable [6,7].

Distributed Temperature Sensing (DTS) technology has proven to be highly effective in the oil and gas industry, where it was first utilized for industrial applications such as energy management and fire detection [8]. Since then, DTS has been implemented in various environmental settings. It has been successfully employed in monitoring lake surface temperatures [9], ground surface temperature variations [10], permafrost distribution in mountains [11], volcanoes [12], scour events [13], and hydrologic systems [14]. In addition to DTS, optic fiber technology is employed in Distributed Acoustic Sensing (DAS), which is utilized for recording earthquake waves and other seismic signals (e.g., [15,16,17]). 

The marine environment presents challenges for the applications of distributed sensing with optical fibers (e.g., deployment, calibration, and operational challenges) [18]. Despite these difficulties, substantial research efforts have been made to explore and utilize DTS technology in various marine-related activities. Several studies have highlighted its potential, including research focused on real-time sea monitoring of oceanic variables [19], monitoring of marine structures [20], and examining seafloor temperature [21,22].

In a short-term pilot study conducted by Oceanus-Lab (Un. Patras) in collaboration with SILIXA Ltd., a DTS wrapped in a frame was deployed over a very productive seabed with intensive and continuous gas seepages in Katakolo harbor (W. Greece). The gas bubbles, with slightly lower temperatures compared to seawater rising from the seabed, were successfully recorded by DTS [23]. Except for this first attempt, the seabed temperature at gas seepage sites has been mainly studied using multi-parametric seabed observatories [24,25,26], ROVs equipped with temperature sensors [27,28], and stationary CTDs [29,30].

The causal relationship between gas emissions from a variety of gas-related marine geological/geomorphological settings (pockmarks, mud volcanoes, gas fields) and earthquakes has been the subject of many multidisciplinary studies using direct and indirect approaches (e.g., in [24,29,31,32,33,34,35,36]). The accumulated knowledge from the investigations so far shows that this relationship is complex and not systematic. Earthquake parameters, monitoring site characteristics, and methodological limitations are some of the factors that have been proposed to explain the lack of consistency between gas emissions and earthquakes.

In the context of this work, seafloor temperature data in a selected shallow area (<30 m) consisting of gas seepage-related settings (pockmark and active fault), located within the pockmark field of Patras Gulf (Western Peloponnese, Greece) [37,38], have been acquired for a period of more than 1.5 years, using a 200 m long DTS system. So far, the Patras Gulf pockmark field has been activated at least two times by two strong earthquakes of Μ5.4 and Μ6.4 in magnitude, on 14 July 1993 [29] and 8 June 2008 [39], respectively. In the case of the 1993 earthquake, temperature anomalies had been recorded within the pockmark field prior to, during, and after the main shock and have been attributed to gas emissions [29]. Although the fluid release from the seabed has been linked to local tectonics, the exact relationship between seabed gas emissions and earthquake activity is poorly known and is based on sparse measurements. However, Judd and Hovland, in their book “Seafloor Fluid Flow”, mentioned that “The pockmarks off Patras, northern Peloponnesus, Greece, represent some of the most spectacular and best-documented events in active pockmarks” (pages 230–231) [40]. In this context, the DTS system has been installed to acquire long-term monitoring of temperature data, to explore the links between gas seepage and seismic activity in the Patras Pockmark Field, and to explore any meaningful underlying relationships.

Establishing data processing and analysis methodological queues, including spectral analysis and incomplete data treatment techniques, are among the main objectives of the present work. Preliminary results on the associations between seabed water temperature fluctuations in the monitored pockmark, metocean conditions, and earthquake activity are also reported and discussed. Although most of the temperature signal variance was significantly correlated to tidal, daily, and annual metocean fluctuations, the residual signal suggested a weak connection to earthquake activity. Though no significant local earthquakes have yet occurred after the DTS installation or during the reporting period, further data acquisition and analysis are ongoing for the following years. However, four significant events exhibiting a 4–8 °C increase in the seabed water temperature above the ambient have been detected and seem to be correlated to local seismicity. The causal link between those temperature events and seismicity is discussed to draw hypotheses that deserve further investigation. 

So far, there has been no other example of the deployment of a DTS system for long-term monitoring of seabed fluid flows via seabed temperature variation, with the exception of the short-term pilot experiment that has been conducted in Katakolo Bay (W. Greece) for gas bubble emission monitoring [23]. The present work represents a first proof-of-concept, evaluating the prospects of DTS for monitoring pockmark activity and building on the maturity of suitable data processing tools and methods.

## 2. The Patras Gulf Pockmark Field

### 2.1. Geographical and Geological Setting

**Figure 1:**
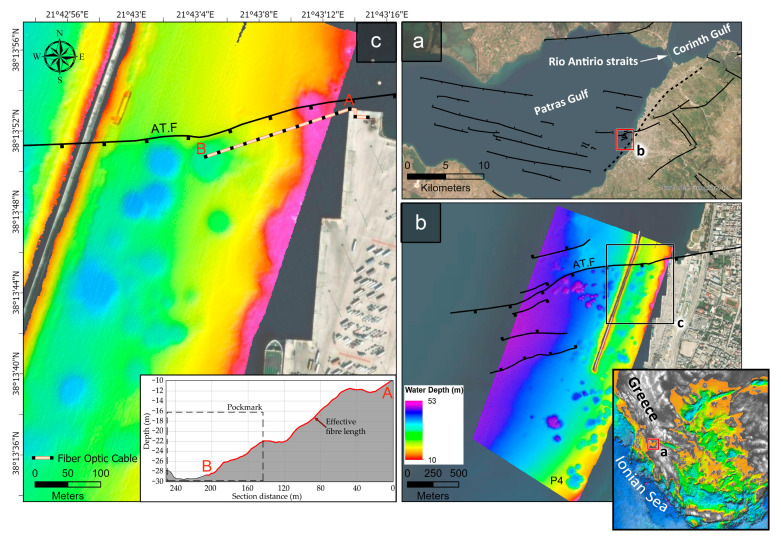
The Patras Gulf pockmark field and the DTS installation. (**a**) Patras Gulf and its main active faults. (**b**) Detailed bathymetry of the pockmark field [38], located around the Patras’s new harbor. (**c**) Detail of the pockmark field and the harbor infrastructure, along with the exact location of the DTS system and its fiber cable. Bathymetric section A–B regards the effective part of the fiber cable.

The Patras Gulf pockmark field is situated in the southern part of the Patras Gulf, near the city of Patras. The gulf is semi-closed, opening into the Ionian Sea on its western side and connecting to the Gulf of Corinth through the Rio-Antirio Strait on its eastern side (Figure 1). The gulf’s topography is shaped by active faults that trend in a WNW-ESE direction [41]. The Patras Gulf pockmark field appears to be located at the intersection of these WNW-ESE, ENE-WSW, and NE-SW fault zones. Although Western Greece is one of the most seismically active areas in the Mediterranean, the Patras Gulf is characterized by lower seismicity compared to the high seismicity of the Rio-Antirio Straits [42]. During the Late Quaternary, the evolution of the Gulf was a consequence of interactions between active tectonic subside (3–10 mm/year), rapid supply of river sediment (2–3 mm/year), and an increment in sea level on a global scale [41,43].

Patras Gulf pockmark field has been the focus of investigations for several years, including seabed and gas-related feature mapping, monitoring of gas seepages, and gas origin identification. The field consists of 115 pockmarks and covers an area of 2.4 km^2^ between the 17- and 45-m isobaths. The pockmarks occupy an area of 0.41 km^2^, approximately 20% of the entire extent of the field (see Figure 1). The pockmarks, based on their morphological characteristics, are distinguished into unit, normal, and composite or complex. The biggest composite pockmark has a diameter of 200 m and a relative depth of 20 m. The Holocene/Pleistocene interface, located 20–25 m below the seabed, is the gas accumulation horizon. The pockmark field is tectonically controlled since a number of W.SW-E.NE trending active faults are running within the field (see Figure 1c) and constitute important pathways for gas seepages [37]. Based on isotopic analyses, the gas is of microbial origin. The seabed in the area of the pockmark field consists of fine-grained sediments ranging from mud to sandy mud and silt [44]. In the last two decades, the pockmark field has been heavily modified by the construction of the Patras new harbor in the same area, and as a result, 23 pockmarks have been totally or partially covered [38], with possible impacts on its ongoing activity.

### 2.2. Pockmark Field Activation and Gas Seepages’ Temperature Imprint

Profiling and side-scan sonar data have documented the activation of the Patras Gulf pockmark field in times of earthquake activity by recording venting gas bubbles from several pockmarks. This finding was observed in the 1993 (M5.4) and 2008 (M6.4) earthquakes. In the 1993 earthquake, of which the epicenter was located 6 km ESE of the field, the seawater temperature was measured using a stationary CTD probe. The acquired data showed that prior to and during the earthquake, the seawater temperature, 10 m above the seabed, increased by about 6.2 °C (from 16.8 to 23.0 °C [29]). The increases in temperature were observed in time snaps and were interpreted as being associated with gas emissions from the field [29]. Moreover, it was observed that at least nine pockmarks were located close to the Ag. Triada Fault (AT.F in Figure 1b,c) and were still venting gas bubbles three to four days after the earthquake. 

In an effort to further investigate the relationship between gas emissions and earthquakes, a multi-parametric observatory, the Gas Monitoring Module (GMM), was deployed inside the largest pockmark of the field in 2004 [26]. The single-frame GMM was landed on the center of the pockmark, with the sensors mounted on its frame, a few centimeters above the seabed. The observatory operated for 201 days and acquired data regarding the physiochemical parameters of the bottom water. During the operation of the GMM, no strong earthquakes happened. However, the GMM recorded more than 60 micro-seepages, showing dissolved methane increases that usually began and ended with changes in the physical properties of seawater. Contrary to the observations in the 1993 earthquake study, the most intense emissions in the 2004 survey were characterized by sudden drops in seawater temperature (T) and pressure. Decreases in temperature were in the order of 0.1–1 °C (up to 1.7 °C) below the ambient T, with all events showing stronger decreases in temperature (ΔT > 0.01 °C within 10 min) associated with decreasing pressure. The authors in [26] suggested that the temperature decreases can be related to the release of gas via bubbling and/or pore-water burst, which would be at a lower temperature than that of ambient seawater. The differences in seawater T changes recorded between the above-mentioned surveying periods of strong (in 1993) and calm seismic activity (in 2004) highlight the complexity of the relationship between seabed water temperature variations, gas venting, and their triggering mechanisms.

However, drops in T associated with gas emissions were also recorded in a close to the studied area, an active field of gas seepages, in the Katakolo Harbour (W. Greece). This area is affected by vigorous gas seepage, with several seeps within a few square meters releasing CH4 continuously but without the formation of any pockmarks. The deployment of the GMM there showed an inverse relation between CH4 concentration in gas seepages and seawater T just above the seabed [45]. This inverse relationship between CH4 and T was recorded in most of the sudden events of CH4 increases, suggesting that gas seepages are characterized by slightly lower temperatures (0.4–0.6 °C) compared to the ambient T.

## 3. Materials and Methods

### 3.1. Monitoring Site Selection and DTS Deployment

The site for the DTS deployment was chosen based on the geological settings in the area and the safety of the system during operation. Based on previously acquired geophysical data [38], a specific pockmark, located at the shallowest part of a pockmark string aligned with the Agia Triada fault (AT.F) inside the Patras harbor, was selected for the deployment of the DTS (Figure 1c). The selected site appears particularly favorable for the DTS deployment and monitoring for the following reasons: (i) the depth is shallow enough (<30 m) and close to the coast to ensure a safe deployment/recovery task; (ii) the fiber cable is running almost parallel and very close to the trace of the active fault of Ag. Triada (AT.F), which has likely been activated in the 1993 earthquake; and (iii) the fiber optic terminates inside a shallow pockmark having a diameter of about 100 m and a relative depth of 22–30 m, in which gas flaring had been recorded in the 1993 earthquake [37]. The fiber had an active bottom length of 200 m, with its last 60 m located inside the pockmark and ending close to its deepest point (see bathymetric section in Figure 1c). 

The SILIXA Ultima-DTS “https://silixa.com/technology/ultima-dts/ (accessed on 19 August 2023)” was installed, known for its fanless design for improved reliability, its increased data storage capacity, and its low energy consumption. The system excels in maintaining precise temperature and sampling accuracy, achieving resolutions of up to 0.01 °C and 25 cm, respectively. Its architecture makes it suitable for distances of up to 10 km and temperatures of up to 600 °C. It has four optical channels, and it can be configured to provide both single-ended and double-ended measurements. This model operates on the fundamental principle of combining Raman-based temperature measurement with Optical Time-Domain Reflectometry (OTDR). By launching a brief pulse of light into the optical fiber, both forward-propagating and Raman backscattered light at distinct wavelengths, known as “Stokes” and “anti-Stokes,” are generated along the fiber’s length. Monitoring the amplitudes of these light rays enables spatial localization using the fiber’s propagation speed. The temperature profile is determined by calculating the ratio of Stokes and anti-Stokes amplitudes, with the former being weakly temperature-dependent and the latter strongly temperature-dependent [46]. The installed system was single-ended, meaning that the far end of the fiber was not connected back to a different channel of the DTS. Assuming the user configuration is optimal (i.e., offset and differential loss calibration have been applied), then the temperature accuracy of the system can be ±0.1 °C plus an additional ±0.1 °C for every 10 °C ambient temperature variation where the system is installed. Offset and differential loss calibration have been performed at the beginning of the experiment and before full deployment of the fiber on the seafloor by making a loop and bringing the cable back close to the DTS. An external Pt100 temperature probe inside a thermally isolated box containing 10 m of the far end of the fiber loop (reference section) was used to determine the slope of the temperature recordings over the fiber length. The difference between the temperatures measured simultaneously by the independent probe and the reference fiber section determined the offset. The acquired calibration offset and slope values were then added to all points of the DTS measurements for the rest of the experiment, with the full length of the fiber placed on the seafloor.

### 3.2. DTS Data Acquisition and Manipulation

**Figure 2:**
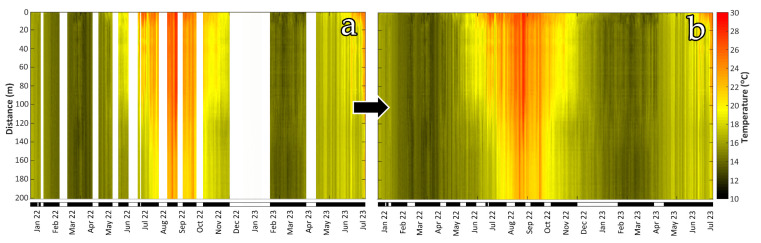
Comparison between the raw (**a**) and the CLEANed (**b**) longitudinally merged time series of the DTS recordings.

The sampling interval of the DTS was set to 0.5 m in the longitudinal dimension and 30 s in the time domain, permitting 400 times and range-averaged temperature measurements per 30 s. An XML file was stored per 30 s, containing the 400 longitudinal temperature measurements for the regarded timeframe. A dedicated MATLAB routine to read the XML files, time-tag them, and sequentially merge all time-averaged longitudinal samples together in a uniform database was created. The dataset considered in the current work refers to a period of 1.56 years (1,613,880 time-averaged samples × 400 longitudinal samples = 645,552,000 actual samples) between December 2021 and July 2023. Due to the inherent difficulties of the installation, corrupted or lost data were frequently induced, mostly due to instrument malfunctions and maintenance activities, resulting in data gaps, as presented in the time vs. fiber-length temperature plot of Figure 2a.

### 3.3. Earthquake Activity and Environmental Data Retrieval

**Figure 3:**
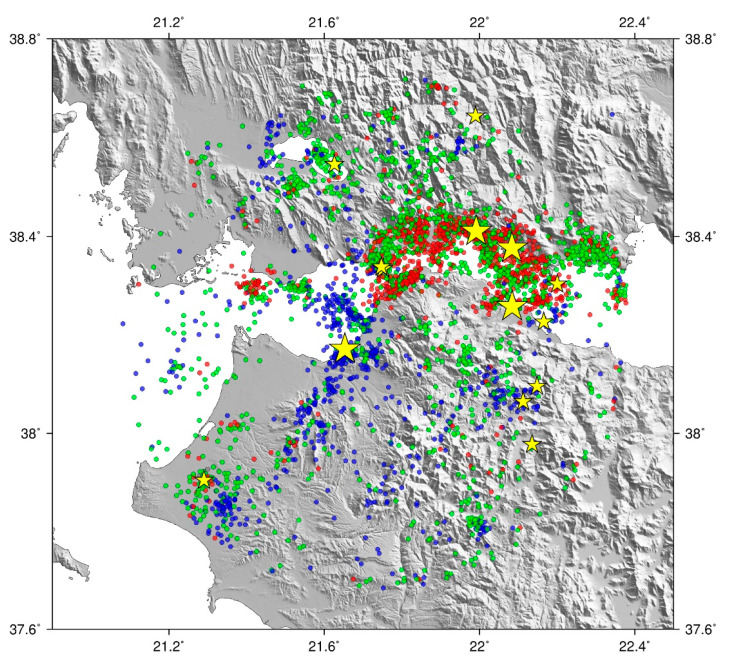
Seismic epicenters in a radius of 60 km around the DTS system for the regarded time period. Red dots refer to earthquakes with hypocentral depths < 10 km, green ones in the range of 10–20 km, and blue ones > 20 km. Small yellow stars indicate earthquakes > M3.5 and bigger ones > M4.

Earthquake source parameters were obtained from the online seismicity catalog of the Institute of Geodynamics at the National Observatory of Athens “https://bbnet.gein.noa.gr/ (accessed on 20 August 2023)” for the regarded time period and a radius of 60 km centered on the DTS location in the new Port of Patras. The Hellenic Seismic Network is particularly dense around the Gulf of Patras, and so it was reasonable to consider the bulletins complete down to 0.1-moment magnitude (M_W_). This dataset contained the date-time, the M_W_, the geographic coordinates of epicenters (Figure 3), the hypocentral depths, and the epicentral and hypocentral distances to the DTS system for all earthquakes in the corresponding period. The dataset selection radius has been set long enough so that any big (>M5) earthquake that has occurred in the far field would be included in the analysis. No such earthquakes occurred in the reporting period, and eventually, only the ones with distances < 10 km were examined.

Meteorological parameters and sea level height measurements were obtained from weather and tide stations located in the premises of the new Patras Harbor, installed in the context of the TRITON (Greece-Italy/Interreg V/A 2014-2020 cooperation) project. Employed environmental parameters eventually contained wind speed and direction, precipitation height, atmospheric temperature, and sea level height, all sampled in a 1-min interval for the regarded time period (1 December 2021–7 July 2023). Although it has been stated that swell has a significant forcing effect on the gas seepage flux due to gas charging, sediment fracture forcing, and pore activation [30], no directional wave time series has been recorded during the DTS experiment. Since the survey site is well protected by a harbor breakwater, it is expected to be slightly influenced by wind-induced wave forcings.

### 3.4. Spectral Analysis with Incomplete Data: The CLEAN Algorithm

The problem of missing values is a common obstacle in environmental time series analysis. There may be various reasons for missing values, for instance, equipment failure, errors in measurements, faults in data acquisition, as well as natural hazards. Whatever the reasons for their existence, missing values constitute a significant problem for environmental applications that require continuous datasets (e.g., [47]). Time series analysis is a common tool in studying environmental processes [48]. Modeling environmental data from a statistical perspective presents the challenge of dealing with a time series that is nonstationary, non-negative, and has a skewed distribution. Likewise, most time-series analysis procedures, like spectral analysis and auto- and cross-correlation, require stationery gap-free time series that also share their samples at common time points. The above data considerations, along with the complex phenomena that control environmental processes, induce great limitations in predicting missing values and explain why no modeling technique has ever exhibited perfect accuracy. Presently, there are a large number of statistical techniques available for dealing with missing values, which can be divided into three categories [49,50]: (1) Listwise/pairwise deletion; (2) imputation-based procedures such as mean, regression, and hot deck; and (3) model-based procedures, which are an active field of research and include among others multiple imputations (e.g., [51], spatial modeling (e.g., [52]), Autoregressive (AR) modeling (e.g., [53,54], Self-Organizing Maps (SOM), Artificial Neural Networks (ANN), and the Expectation Maximization (EM) algorithm. Most of them require neighbor stations to search for or assume a good correlation between their observations to predict missing values. In the present study, no prior knowledge exists on the temporal patterns of the seabed water temperature, and so most of the above methods are not applicable. Moreover, the present dataset is expected to be strongly influenced by day-night and seasonal heat flux equilibrium processes, affecting water temperature, while the tidal currents might also input tidal periodicities to the signal. The above signifies that a missing-values prediction method based on spectral analysis would most likely make accurate predictions for most of the data variance, but it may not account for any random irregularities, such as extreme meteorological events, other anthropogenic factors (e.g., marine traffic), or events related to earth dynamics (i.e., earthquakes and sediment dynamics). Missing value imputation methods based on the frequency domain are either using the Fourier [55,56,57] or the wavelet (e.g., in [58]) spectrum of the time series.

The CLEAN algorithm has been chosen in this work for filling in missing data. It is an effective tool for predicting missing values in both stationary and nonstationary time series using spectral analysis and was introduced by [57], with applications of proven significance in seismology [59] and tidal analysis [56]. The main advantages of the algorithm are that it removes spectrum artifacts related to missing data, provides stable spectral peaks [55], and does not require a formal statistical test [60]. Furthermore, Ref. [61] showed that the CLEAN algorithm can effectively recover most of the lost information even for significantly fewer data points. In the CLEAN algorithm, a raw frequency spectrum, otherwise called dirty, is calculated using DFT, which contains real peaks and side lobes (aliases) caused by the contribution of the spectral window. This dirty spectrum is then iteratively cleaned. The largest spectral peak is found, and its side lobes are subtracted from the original dirty spectrum. In the next iteration, the largest peak is detected in the residual dirty spectrum and is compensated for. The procedure is repeated until a defined number of iterations is reached, finally composing the final spectra (from now on called the CLEANed one). In this work, the number of iterations was set to 3000, which was found to be enough for the spectra to converge.

The Inverse Discrete Fourier Transform (IDFT) was then applied to reconstruct the time series, using a predefined time step interval so that the new ones are stationary and their missing values are simulated. Usually, the time step interval for the IDFT is defined as the minimum time difference between the samples. However, according to the Nyquist sampling criterion, the output time series should have half (or less) the sampling frequency of the raw ones. Following on from this, in the present study, the spectral analysis was performed on the basis of double the sampling interval, producing a completed time series of a minute’s time step interval.

To apply the CLEAN algorithm to the available temperature time series, we used the [59] computational implementation for MATLAB, modified to accept user-specified time step intervals. The CLEAN algorithm was applied per longitudinal sample, producing 400 reconstructed time series (per 0.5 m range interval), containing 806,940 time samples each (per 1 min), and reaching 322,776,000 temperature samples in the dataset. The prediction accuracies of the reconstructed time series were, in all cases, higher than 99.8%, as evinced by estimating the Pearson correlation coefficient between them and the raw data where they existed. Thus, the reconstructed time series retained the vast majority of the raw dataset information and was considered suitable for performing any further statistical analyses. However, to avoid even insignificant mispredictions of the raw temperature data, they have been re-inputted to the simulated ones so that only missing data were predicted and filled in. A comparison between the raw and the gap-free, longitudinally merged time series is presented in Figure 2a,b.

### 3.5. Data Preparation and Statistical Treatment

The final dataset comprised both stationary and nonstationary time series, with the former being the CLEANed DTS temperature measurements and the meteorological ones, and the latter the earthquake magnitude ones. Performing statistical comparisons between stationary time series requires common time intervals and time sampling points. The CLEANed DTS and the meteorological time series already had an interval of 1 min, so no further elaboration was needed.

A Comparison between the DTS temperature and meteorologic time series has been performed via the windowed cross-correlation function, as implemented for MATLAB in [62]. This splits the signals into overlapping segments of a defined duration and estimates their cross-correlation up to a maximum prespecified time lag. This is particularly useful in environmental variables that might respond at different speeds to underlying triggers or that are sequentially triggering one another.

To specify events of irregular temperature time series, any outlier has been detected using the interquartile range (IQR), i.e., the range of the middle half of the statistical distribution of the dataset, and the outliers were defined as any values that fall outside this range. IQR is the range between the first and third quartiles (IQR = IQ3 − IQ1), while the highest bound of regular data, over which any sample is considered an outlier, is estimated as Q3 + 1.5 × IQR [63]. Any temperature even higher than this threshold has been indicated as an event (E#) and was compared against the magnitude, depth, and epicentral distance of the earthquakes that occurred during and around the corresponding time periods (see Section 4.4).

## 4. Results

### 4.1. DTS Data Screening: Date-Time and Depth Dependencies

**Figure 4:**
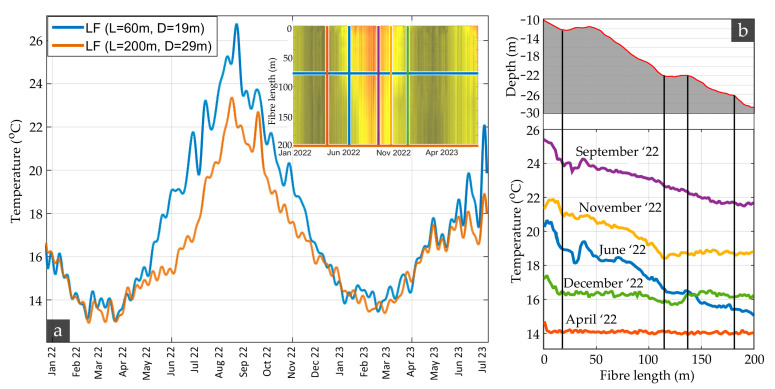
(**a**) Comparison between a temperature time series outside the pockmark (depth = 19 m; fiber length = 60 m) and one to its deepest point (depth = 29 m; fiber range = 200 m). (**b**) Comparison of temperature profiles along the fiber for a selection of five different months in 2022.

Comparison between seabed water temperature time series outside the pockmark (depth = 15 m; fiber length = 60 m) and close to its deepest point (depth = 29 m; fiber range = 200 m) (Figure 4a) revealed similar temporal trends, with the former exhibiting higher extremes during the summer. 

To explore the effect of the normal water column temperature change with depth, a comparison between DTS profiles across the entire fiber cable is visualized in Figure 4b for a selection of five different time points. A strong correlation between depth and seabed water temperature is evident, especially during the summer and autumn. This is particularly apparent in the November, December, and June profiles of Figure 4b, where clear breakpoints are apparent around the 120 m fiber length point. This is expected due to the normal seasonal temperature gradient and thermocline formulated during those seasons in the area (summer temperature profiles can be found in [29]).

### 4.2. Spectral Analysis: Separating Background Environmental Components from Anomalous Events

**Figure 5:**
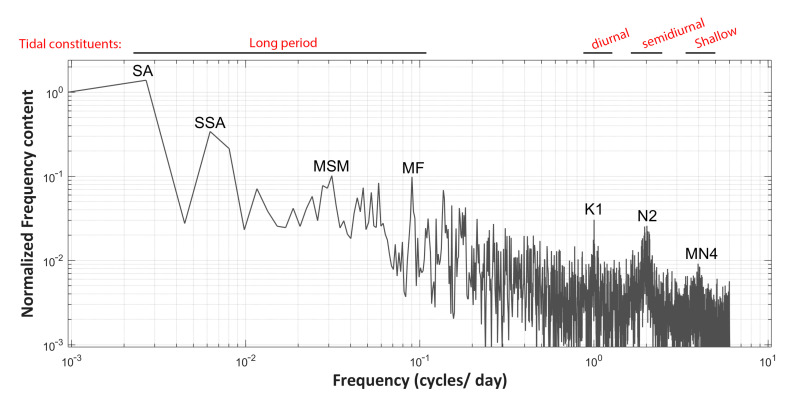
The CLEANed frequency spectrum of the seabed water temperature time series close to the pockmark center (200 m longitudinal sample along the fiber). The major tidal harmonic constituents detected are indicated. Both the x and y axes are on a logarithmic scale.

**Figure 6:**
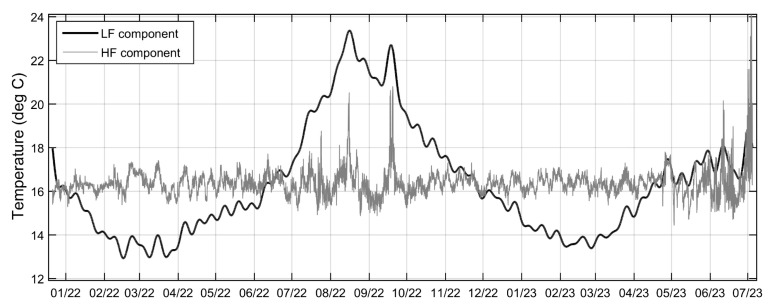
Combined time series plot of the low frequency (LF) and high frequency (HF) components of the seabed water temperature signal at the end of the fiber (200 m longitudinal sample). LF and HF correspond to low and high-frequency components of the CLEANed signal, below and above 1 cycle/day frequency thresholds, respectively.

The CLEAN method has been applied to all longitudinal time series to compensate for the missing values and allow for spectral and time series analysis. In Figure 5, the CLEANed frequency spectrum of the temperature time series close to the pockmark’s deepest point (200 m longitudinal sample along the fiber) is presented. All major harmonic constituents are found to be tidal, and more specifically: (a) Long period ones, including solar annual (SA), solar semiannual (SSA), lunisolar monthly (MSM), and lunar fortnightly (MF); (b) the lunisolar diurnal (K2); (c) the lunar semidiurnal (N2); and (d) the higher harmonic ones, represented by the shallow water quarter diurnal (MN4). Those spectral components explain the vast majority of the data variance, offering the opportunity to investigate other irregular events in the signal reconstructed via the residual spectra. Thus, to better visualize and statistically elaborate on the dataset, every longitudinal time series has been decomposed using bandpass filters into two-time series, a high frequency (HF) and a low frequency (LF), based on a frequency threshold of 1 cycle/day (see Figure 6). Decomposition of those time series has been performed via inverse Discrete Fourier Transform (iDFT) application to the CLEANed spectrum, cropped on the above-specified spectral band. Any unusual event related either to earthquakes or to other irregular anthropogenic activities that involve ground vibration or sediment resuspension (e.g., marine traffic) is expected to be clearly visible in the HF residual time series. In Figure 6, a combined time series plot of the low-frequency (LF) and high-frequency (HF) components of the temperature signal at the end of the fiber cable (200 m longitudinal sample) is presented. All longitudinal time series have exhibited the same seabed water temperature irregularities, and only the deepest time series are further analyzed in this work.

### 4.3. Correlation to Environmental Datasets

**Figure 7:**
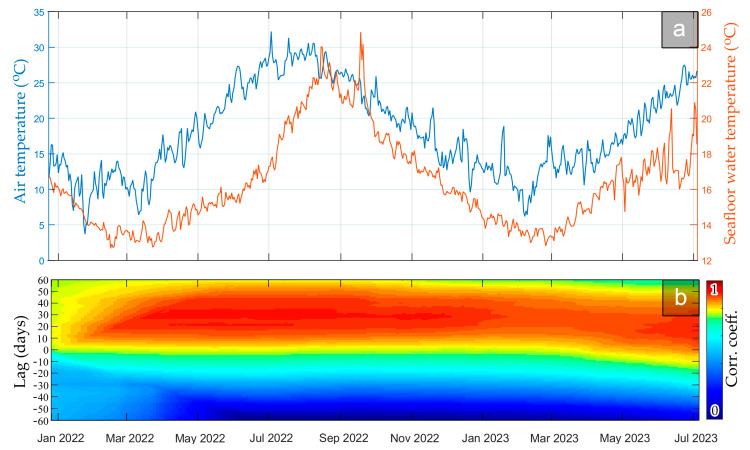
(**a**) Combined time series plot of the atmospheric air versus the seabed water temperature during the monitoring period. The seabed water temperature signal corresponds to the deepest point of the DTS fiber (200 m longitudinal sample). (**b**) Windowed cross-correlation plot between the above time series, indicating a 20–30 days’ time lag between them.

In Figure 7a, the atmospheric temperature is compared against the seabed water temperature in the pockmark during the monitoring period. Application of the windowed cross-correlation exhibited a 20–30 day time lag between them, indicating that the seafloor water temperature reaches its maximum about a month later than the atmosphere’s. This is normal, as the heat flux equilibrium between solar radiation, longwave radiation, latent heat, and sensible heat, combined with vertical mixing and horizontal advection, allows heat storage to be maximized in the water column later than in the atmosphere.

No significant correlation between the DTS data and any other meteorological parameter (precipitation and wind) has been made evident. An in-depth examination of the correlation between DTS and sea surface height indicated no other findings than the tidal contribution, which is otherwise revealed through spectral analysis in Section 4.2. This tidal dependency of seafloor water is likely owed to the strong tidal currents applying in the Gulf of Patras (see [64]), bringing waters of different temperatures either from the Corinth Gulf or the Ionian Sea in a semidiurnal fashion. 

### 4.4. Association with Earthquake Activity

**Figure 8:**
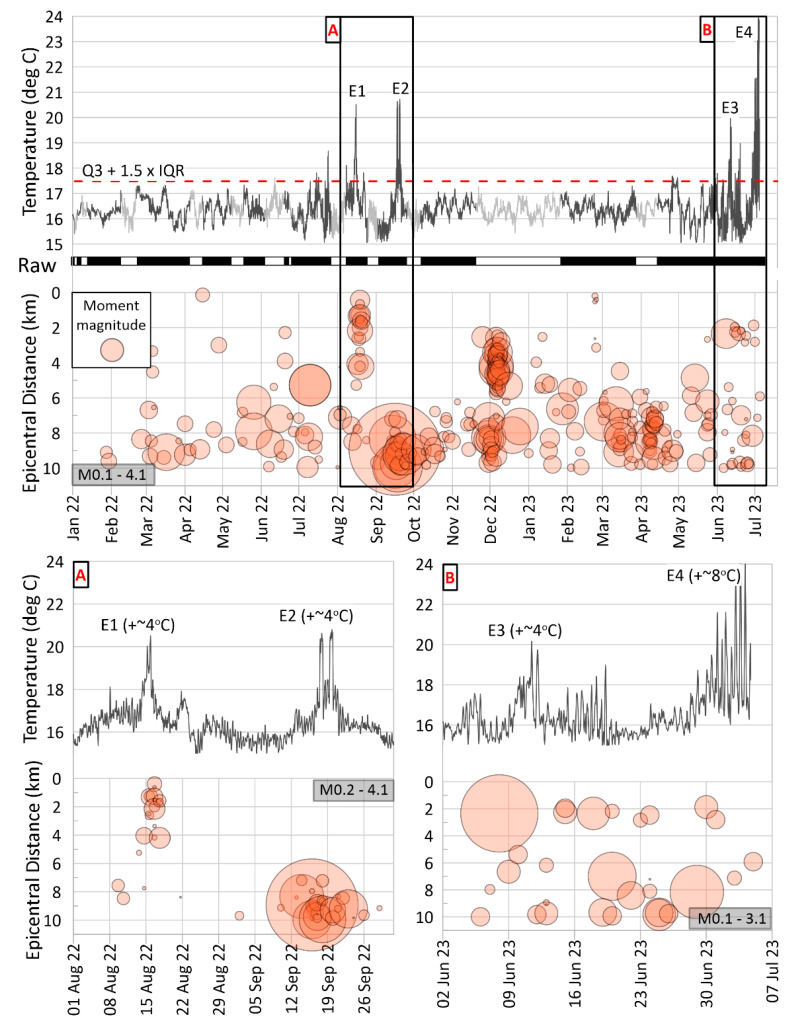
Comparison between the seabed water temperature in the pockmark (200 m longitudinal sample) and the moment magnitude of each earthquake versus its epicentral distance to the DTS during the monitoring period. In the lower half, two magnified periods (**A**,**B**) are shown, where abnormal temperature events (E1–E4) occur. Those have been specified as outliers through the interquartile range (IQR) formula (red dashed line in the upper-temperature plot). Black/white bar between the upper plots corresponds to the actual (raw = black) versus the simulated (missing = white) data, while simulated data are presented in grey color. The seismic moments are represented as circles with a radius relative to the seismic moment, scaled by the 2/3 rational power. The magnitude ranges are indicated in grey boxes per seismic plot.

The motivation for conducting the experiment described in this paper was, as previously mentioned, the observation of a pockmark field activation through the seabed water temperature variations in relation to instances of earthquakes in the region. One of the principal scientific inquiries we aim to address through the ongoing data collection is whether there exists a correlation between pockmark field fluid flow events and seismic activity, and if so, what the underlying physical mechanism for this correlation might be. At present, the time period of the available data is not sufficiently long to support a statistically robust investigation of this question. However, in Figure 8, we initiate a preliminary comparison between the observed seabed water temperature anomalies and the seismic activity below and around the pockmark field, with the goal of identifying evidence that could either bolster or challenge the hypothesis of a correlation.

In Figure 8, we present the seabed water temperature close to the center of the pockmark (200 m longitudinal fiber cable sample) for the examined period. Temperature anomalies have been objectively identified through standard statistical analysis regarding the calculation of the Interquartile Range, as described in Section 3.5. These identified outliers are indicated in Figure 8 by black frames and are assigned code names E1–E4. These thermal events last about 4 to 5 days. Below the temperature plot, a black/white bar is displayed, demarcating intervals of actual (black) versus simulated (white) data, while the simulated data have been indicated grayer to avoid visual confusion in the comparison.

In each sub-plot of Figure 8, we illustrate the temporal evolution of seismic activity throughout the observation period within a 10 km epicentral distance radius of the DTS. Each circular symbol represents a seismic event, with the size of the circle corresponding to the seismic moment of the respective earthquake. The vertical axis denotes the epicentral distance measured from the DTS. The highest recorded earthquake magnitude near the field during the examined time frame was M4.1. Other plotted events exhibit smaller magnitudes in the range of M0.3–M3.7. Microseismic activity is generally dispersed in time and is observed at distances exceeding 5 to 6 km from the DTS point.

**Figure 9:**
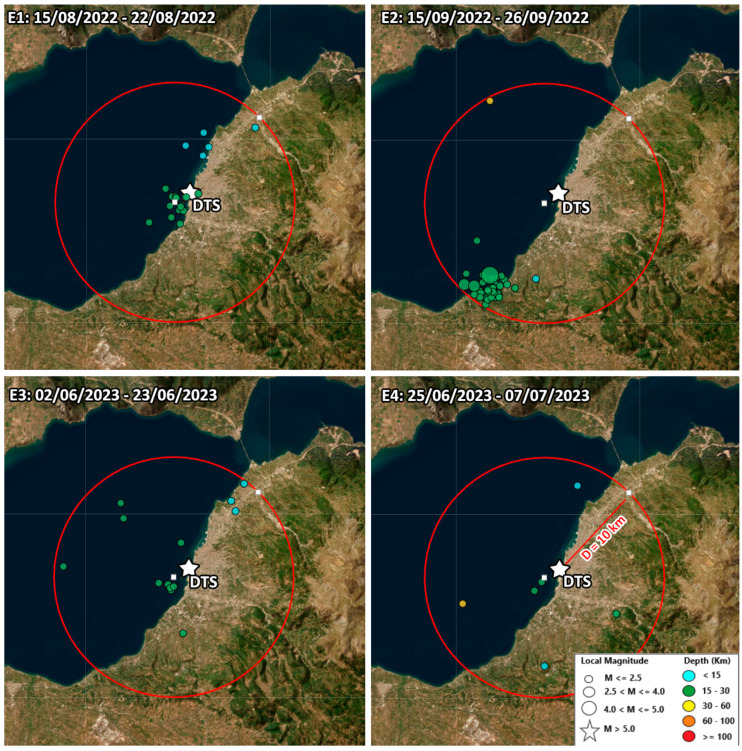
The seismicity in the Patras Gulf area during the four detected seabed water temperature high events (E1–E4, see Figure 8). Dots represent earthquake epicenters during the time period indicated in the upper right corner of each sub-map. Dot colors indicate the focal depth of each earthquake, while dot sizes correspond to their local magnitude (in the Richter scale).

However, specific clusters of seismic events are discernible. Two of these clusters synchronize with two temperature outliers (designated as frame A in Figure 8), while one additional cluster occurred during a period with no available DTS data (1–10 December 2022). Another cluster, spanning an extended time frame, is observable toward the conclusion of the plotted time span (identified as frame B in Figure 8). The geographic representation of these clusters is depicted in map views in Figure 9.

## 5. Conclusions/Discussion

A DTS system consisting of a 200 m long fiber optic cable was deployed inside a pockmark and along an active fault situated in the Patras Gulf pockmark field for 1.56 year. The entire field and, in particular, the monitoring site have experienced at least two activations due to strong earthquakes in the past, as indicated by the intense gas emissions after the main shocks [29,37,38,39]. One of the main objectives of our work was to present an experimental methodology for high-resolution seafloor water thermal profiling (DTS) and establish a data processing scheme for the analysis of DTS, metocean, and seismic datasets to find possible links between them. Monitoring the underlying relationships between seismic activity and gas escapes through the continuous temperature recordings at the seabed was also one of the main objectives of the experiment. This experiment in the Patras Gulf pockmark field is proof of concept that the DTS installation produces meaningful results and, given that it maintains in time, it may constitute the first long-term active pockmark seabed temperature monitoring system through DTS mobilization. In this context, the ultimate scope of this effort is to suggest cost-effective monitoring systems, such as DTS, for use in shallow coastal environments and tackle challenges associated with the dynamic nature of the underwater environment, including deployment problems, sustainability, and reliability of data collection.

Although during the last three decades, enough multidisciplinary relevant surveys have been carried out, relatively little is known about the role of earthquakes in triggering gas seepage and about the link between earthquakes and gas seepages in a variety of gas-related settings (faults, pockmarks, mud volcanoes). The lack of consistency of that link can be explained by earthquake parameters (magnitude, hypocentral depth), tectonic and sedimentary settings, and, in the case of long-term seabed multi-parametric observatories, by the monitoring site characteristics and the methodological limitations. The authors of [65] suggested that this lack of consistency may also originate from monitoring site effects induced by the seismic energy radiation pattern.

The absence of significant earthquakes during any time-limited monitoring period using multiparameter observatories introduces uncertainty to the study of the causal link between seismic activity and gas seepages. A benthic observatory equipped with a seismometer was deployed at the NAF (North Anatolian Fault), but no significant local earthquakes (>M3.6) occurred during the monitoring period (161 days) [24]. Similarly, in [26], a benthic multi-parametric observatory was deployed, for the first time ever, inside an active gas-bearing pockmark at the southern end of the Patras Gulf pockmark field. No significant earthquake occurred during this monitoring period (201 days), and the link between local low-magnitude seismicity and gas emission events was not considered worth studying.

In our case, no significant earthquakes occurred during the DTS monitoring period. The coincidence of local earthquakes within a radius of 10 km with temperature increase events at the monitoring site is considered not systematic. Although the link is rather occasional, meaningful associations have been observed in four cases. Four temperature increase events (E1–E4) seem to have a possible relationship with swarms of low to moderate-magnitude local earthquakes (see Figure 8). Three of them (E1, E2, and E3) show a remarkably similar pattern: a 4 °C temperature increase with a duration of 4–5 days each, with their peaks almost synchronous to the seismic events. During those thermal events, no bottom temperature difference was recorded between the interior and exterior parts of the pockmark, possibly suggesting the contribution of Ag. Triada active fault to the increase in the seafloor water temperature. This is likely, as the fault has been activated at least twice in the past by earthquakes releasing significant amounts of gas.

If we hypothesized that these thermal events are related to local low seismicity, then these findings are in accordance with the observations in [29]. Prior to, during, and after the 1993-M5.4 earthquake, the temperature increased anomalously three times at a monitoring CTD station located 10 m above the seabed. In the post-earthquake period, at least nine pockmarks continued venting gas bubbles, as evinced through hydroacoustic data. The gas flares were mainly concentrated in the northern part of the field at the pockmarks and along the Agia Triada fault, close to where the current DTS system has been installed. Those temperature increases have been interpreted as related to gas seepages [26,29]. The release of fluids from the seabed and the subsequent increase in bottom water temperature have been reported elsewhere. The short-term temperature increase has been linked with the release of warm, CH4-rich fluids expelled from the sediments as triggered by short-duration seismic events (SDEs) [66]. Transient fluctuations in the intensity of fluid flow from the seafloor, occasionally triggered by earthquake swarms, have also been shown to alter bottom water temperatures locally [67].

An interesting hypothesis supporting that the findings of the present study are in accordance with those in [29] could be outlined for the E2 event. While this earthquake is also relatively minor in magnitude (cluster M4.1, Figure 8A) and it occurred at a distance of ~10 km from the DTS, it was distinctly felt in the vicinity and could have conceivably perturbed the mechanically weak sediments within the pockmark field. Worth highlighting is the depth at which this seismicity is occurring—approximately 65% of the events are positioned within the depth range of 19–25 km. This contrasts with the shallower depth of the extensively studied microseismicity of the western Gulf of Corinth (e.g., [68]). However, the authors of [69], in their study of the aftershock sequence resulting from the impactful M5.4 earthquake near Patras in 1993, similarly proposed earthquake foci at relatively greater depths (14–22 km) and observed a spatial distribution of aftershocks aligned with the NW-SE direction. A compelling conjecture arises that there is a northwestward continuation of the fault associated with the 1993 seismic activity. This possibility is in accordance with the prior connection established between the 1993 seismicity and the activation of the pockmark field [29]—even preceding the mainshock of the sequence, since in both earthquakes (M4.1—2022) and (M5.4—1993), events of increased seabed water temperature have been recorded. This emphasizes the imperative need for sustained multidisciplinary monitoring of the pockmark field and for systematic investigation of the potential interconnections among the measured parameters.

Safer conclusions could only be drawn after a considerable amount of time has been spent monitoring the pockmark. The experiment is still ongoing, and so an in-depth validation is intended to be realized in the following years. The herein implemented methodological que, and especially the incomplete data treatment and the spectral decomposition of the signal into LF and HF signals, will form the basis of any future data analysis efforts. Such efforts are also expected to be further enriched with the statistical treatment of the earthquake list of events to derive a stationary time series of seismic energy suitable for a quantitative correlation to temperature. Regarding the DTS installation, an extension of the fiber optic cable should also be considered so that more pockmarks are monitored while a crossing of the AT.F. should be achieved. Separate monitoring in a control area, far from the field, should be considered to acquire background ambient temperature signals both during periods of increased seismic activity and during periods of normal activity.

## Data Availability

Data are available on request.
